# Association Between the Expression of Cytochrome P450 and Glutathione S-transferase Enzyme and Antrochoanal Polyp Pathogenesis

**DOI:** 10.1055/s-0044-1801320

**Published:** 2025-05-29

**Authors:** Mehmet Gökhan Demir, Sedat Aydın, Serpil Oguztuzun, Kayhan Basak

**Affiliations:** 1 Department of Otorhinolaryngology and Oral and Maxillofacial Surgery, Istanbul Medical School, Istanbul University, Istanbul, Turkey; 2 Department of Biology, Kırıkkale University, Kırıkkale, Turkey; 3 Department of Pathology, Dr. Lutfi Kirdar Kartal Training and Research Hospital, Istanbul, Turkey

**Keywords:** antrochoanal polyp, allergy, antioxidant, glutathione S-transferase, cytochrome P450

## Abstract

**Introduction** Antrochoanal polyp, which is a kind of smooth-surfaced single nasal polyp, is commonly present in cases of nasal obstruction. The pathogenesis of polyp formation is still unclear, but allergy is supposed to be a cause.

**Objectives** To investigate the expression levels of antioxidant enzymes in antrochoanal polyp tissue.

**Methods** The antrochoanal polyp group was composed of 23 patients who were diagnosed microscopically, and the control group was composed of 38 healthy patients. The sample of the control group was taken from the inferior turbinate mucosa by punch biopsy under general anesthesia, and the antrochoanal polyp sample was collected from sinus surgery. The cytochrome P450 (CYP) and glutathione S-transferase (GST) expressions of the groups were investigated under microscopy and scored by senior pathologists.

**Results** The antrochoanal polyp group had statistically less expression of CYP family 1 subfamily A member 1 (CYP1A1) than the control group ( *p* < 0.05). Moreover, GST Pi 1 (GSTP1), GST Mu 1 (GSTM1), and GST Alpha 1 (GSTA1) expressions were not different between the groups ( *p* > 0.05).

**Conclusion** Allergy and chronic inflammation are postulated reasons for antrochoanal polyp formation, but, according to our results, we could not detect any relation between antrochoanal polyp formation and GST expression in tissue. However, the decreased level of CYP1A1 expression in the antrochoanal polyp group may be related with the pathogenesis of the antrochoanal polyp formation.

## Introduction

Antrochoanal polyps (ACPs) grow from the mucous membrane of the maxillary sinus, passes most commonly through the accessory ostium of the maxillary antrum, then spreads through the posterior part of the nasal cavity and prolapses through the choana. 1 2 3 They have been first described in 1906 and represent 4 to 6% of all nasal polyps, being 2 times more common in male patients. In childhood, the incidence is reported to be 33%. 4 The most common symptom is unilateral nasal obstruction. Additionally, ACP can be presented with rhinorrhea, bleeding, snoring, halitosis, headache, postnasal drip, and anosmia. Endoscopic nasal examination reveals a single polyp arising from the middle meatus aligning with the nasopharynx. Histologically, ACP is a kind of inflammatory polyp covered with ciliated cylindrical epithelium. Both plasma cell and eosinophils infiltration can be detected in the edematous loose connective tissue with increased vascularity. Because of these histopathologic findings, allergies limited to the maxillary sinus or inflammation are postulated to be the reason for ACP formation, although the definite reason is still unknown. 5

Recent reports claimed that reactive oxygen species (ROS) production is related to nasal polyp generation. 6 7 8 Reactive oxygen species and reactive nitrogen species, such as free oxygen radicals (O _2_^-^ ), hydrogen peroxide (H _2_ O _2_ ), and nitric oxide (NO), are known to cause progressive cell injury, and, with the oxidative stress occurrence, they damage normal tissues. 9 The main source of ROS products are the eosinophils, neutrophils and lymphocytes found in the non-allergic polyp tissue ^6^ . The antioxidant system of a healthy person utilizes the ROS and its products efficiently ^8^ . Glutathione S-transferase (GST), which is one of the key enzymes in the antioxidant system, is involved in the detoxification of several endogenous- or exogenously derived electrophiles and metabolites and the activation of the antioxidant system. 8 10 11

Cytochrome p450 (CYP) is a kind of super enzyme that has a significant role on both the oxidation and reduction reaction of various cellular compounds. 12 13 It acts on the metabolism of endogenous subtracts, environmental pollutants, and carcinogens. 12 Cytochrome p450, which is the most important enzyme in phase-1 bioactivation reactions in cells, can contribute to activation of precarcinogens and lead to unwanted response at the tissue level. 12 13

The expressions of CYP and GST enzymes in ACP tissue have not been investigated before. The impact of this antioxidant enzyme on tissue level is unclear as we mentioned before. 5 In the present study, we investigate the CYP and GST enzyme expressions in ACP tissue and compare them with those in normal nasal mucosa.

## Methods

The current study is prospective, cross sectional, and control based, and it was conducted in our hospital between 2012 and 2014. The study group consisted of 23 patients diagnosed with ACP confirmed by the pathologist. The diagnosis of the ACP was done with a nasal Hopkins 0-degree, 4-mm rigid endoscope (Karl Störz SE & Co KG, Tuttlingen, Germany), and the polyp tissue was sampled by punch biopsy. The final diagnosis was achieved by the senior pathologist. All participants in the study group were subjected to computer tomography investigation and underwent endoscopic sinus surgery. The polyps, which were collected from the surgery, were examined for GST and CYP enzyme expressions. The study group was also divided into two groups according to smoking habits. Patients with previous nasal surgical history, sinonasal infection or neoplasm, asthma, and any allergic disease were excluded from the study. The information of the study group was included in Table 1 .

**Table 1:** Patients showing expression of CYP and GST isoenzymes in antrochoanal polyp tissues

		CYP1A1	GSTM1	GSTP1	GSTA1
	N	n (%)	n (%)	n (%)	n (%)
**Antrochoanal polyp**	23	5 (21.73)	16 (69.56)	20 (86.95)	14 (60.86)

Abbreviations: CYP, cytochrome P450; CYP1A1, cytochrome P450 family 1 subfamily A member 1; GST, glutathione S-transferase; GSTA1, glutathione S-transferase Alpha 1; GSTM1, glutathione S-transferase Mu 1; GSTP1, glutathione S-transferase Pi 1.

The control group was composed of 38 volunteer healthy people who had not got any sinonasal polyp pathology. The control sample was collected by punch biopsy from the anterior border of the inferior concha during septoplasty operation under general anesthesia and sent to the senior pathologist for further investigation. The demographic information of the control group can be seen on Table 1 .

The Mann-Whitney U test was used to compare the immunoexpression values of the tested molecules between the experimental and control groups, and the Pearson's correlation test was used to test the statistical association between the expression of the molecules. Statistical analysis was performed with the MINITAB14 computer software (Minitab, LLC, State College, PA, USA), and a *p* -value < 0.05 was considered as significant.

*Immunohistochemical staining:* The tissues were fixed in 10% buffered formalin and embedded in paraffin blocks. Sections 4-µm thick were cut, and one section was stained with hematoxylin and eosin to observe the tissue morphology. For immunohistochemistry, endogenous peroxidase activity was blocked by incubating the sections in 1% hydrogen peroxide (v/v) in methanol for 10 minutes at room temperature (RT). The sections were subsequently washed in distilled water for 5 minutes, and antigen retrieval was performed for 3 minutes using 0.01 M citrate buffer (pH 6.0) in a domestic pressure cooker. After being washed in distilled water, the sections were transferred to a 0.05 M Tris-HCl (pH 7.6) solution containing 0.15 M sodium chloride (TBS). The sections were incubated at RT for 10 minutes with super block (SHP 125) (ScyTek Biotech Life Sciences Laboratories, Logan, UT, USA) to block nonspecific background staining. The sections were then covered with the primary antibodies diluted 1:650 for anti-GST Pi 1 (anti-GSTP1), 1:400 for anti-GST Mu 1 (anti-GSTM1), 1:300 for anti-GST Alpha 1 (anti-GSTA1), and 1:400 for anti-CYP family 1 subfamily A member 1 (anti-CYP1A1), in TBS at 4 ^o^ C overnight. Anti-CYP1A1 (monoclonal, sc-20772) and anti-GSTM1 (polyclonal, ab-113432) Abcam Limited, Cambridge, United Kingdom); anti-GSTA1 (monoclonal, sc-100546) (Santa Cruz Biotechnology Inc., Dallas, TX, USA); and anti-GSTP1 (polyclonal, PA1590) (BOSTER Biological Technology., Ltd., Pleasanton, CA, USA). After being washed in TBS for 15 minutes, the sections were incubated at RT for biotinylated link antibody (SHP 125) (ScyTek Laboratories, USA). Then, the treatment was followed with Streptavidin/HRP complex (SHP 125) (ScyTek Laboratories, USA). Diaminobenzidine was used to visualize peroxidase activity in the tissues. Nuclei were lightly counterstained with hematoxylin, and then the sections were dehydrated and mounted. Both positive and negative controls were included in each run. Positive controls consisted of sections of liver tissues for GSTM1 (glutathione S-transferase M1) and GSTA1 (glutathione S-transferase A1), lung tissues for GSTP1 (glutathione S-transferase P1), gall bladder tissues for CYP1A1 (cytochrome P450 1A1). For negative controls, TBS was used in place of the primary antibody.

Light microscopy of immunohistochemically stained sections was performed by a pathologist and a biologist, who were unaware of the patients' clinical information. Distribution, localization, and characteristics of immunostaining were recorded. Brown color in cytoplasm and/or nucleus of epithelial cells was evaluated as positive staining. Scoring was also performed by observers unaware of the patient data. Scoring differences between observers was resolved by consensus. For each antibody, the intensity of the reaction—negative (-), weak (1 + ), moderate (2 + ), or strong (3 + )—was determined in order to describe the immunoreactions. 14

The study was approved by our institutional ethical committee with 2009/005 protocol number.

## Results

The study population was composed of 23 patients with ACP and 38 control patients without any symptoms. Additionally, the ACP and control subjects were divided into two groups depending on smoking habits. Both groups were identical in terms of demographic information ( *p* > 0.05) ( Table 1 ). In Table 2 , protein expressions of GST and CYP enzymes in ACP and control tissues and their statistical differences are given. According to our results, the protein expressions of GST and CYP enzymes in smokers and non-smokers are statistically not different. So, we have not detected any relation between ACP formation and smoking habits ( *p* > 0.05). ( Table 3 ).

**Table 2:** Comparison of the CYP and GST isozyme expression between antrochoanal polyp and control patient.

	ACP N = 23	Control N = 38	*R* ;* *p* -value**
**CYP1A1**	0.30 ± 0.13 ^a^ (0–2) ^b^	0.68 ± 0.11 ^a^ (0–2) ^b^	0.44 **0.0407**
**GSTM1**	0.87 ± 0.15 ^a^ (0–2) ^b^	0.76 ± 0.11 ^a^ (0–2) ^b^	1.14 0.5973
**GSTP1**	1.43 ± 0.15 ^a^ (0–2)	1.21 ± 0.10 ^a^ (0–2) ^b^	1.18 0.1829
**GSTA1**	0.70 ± 0.13 ^a^ (0–2) ^b^	0.76 ± 0.10 ^a^ (0–2) ^b^	0.92 0.6824

**Abbreviations:** ACP, antrochoanal polyp; CYP, cytochrome P450; CYP1A1, cytochrome P450 family 1 subfamily A member 1; GST, glutathione S-transferase; GSTA1, glutathione S-transferase Alpha 1; GSTM1, glutathione S-transferase Mu 1; GSTP1, glutathione S-transferase Pi 1.

**Notes:** The staining scores were calculated based on the sum of the staining intensity of positively stained antrochoanal polyp tissues and control tissues. Staining intensity was graded as: 0 for no, 1 for weak, 2 for moderate, and 3 for strong. Differences in CYP and GST isoenzymes expressions between antrochoanal polyp tissues and control tissues were examined by the Mann-Whitney U test with 95% confidence level. ^a^ Mean ± standard error of the mean; ^b^ minimum and maximum staining intensity; *ratio of antrochoanal polyp tissues and control tissues; **Values of *p* < 0.05 were considered statistically significant.

**Table 3:** Comparison of the CYP and GST isozyme expression between antrochoanal polyp in smokers and non-smokers

	Smoker N = 13	Non-smoker N = 15	*R* ;* *p* -value**
**CYP1A1**	0.40 ± 0.15 ^a^ (0–2) ^b^	0.48 ± 0.11 ^a^ (0–2) ^b^	0.84 0.3407
**GSTM1**	0.82 ± 0.12 ^a^ (0–2) ^b^	0.78 ± 0.11 ^a^ (0–2) ^b^	1.12 0.5793
**GSTP1**	1.33 ± 0.17 ^a^ (0–2) ^b^	1.21 ± 0.10 ^a^ (0–2) ^b^	1.18 0.2129
**GSTA1**	0.71 ± 0.13 ^a^ (0–2) ^b^	0.70 ± 0.10 ^a^ (0–2) ^b^	0.92 0.6924

**Abbreviations:** ACP, antrochoanal polyp; CYP, cytochrome P450; CYP1A1, cytochrome P450 family 1 subfamily A member 1; GST, glutathione S-transferase; GSTA1, glutathione S-transferase Alpha 1; GSTM1, glutathione S-transferase Mu 1; GSTP1, glutathione S-transferase Pi 1.

**Notes:** The staining scores were calculated based on the sum of the staining intensity of positively stained antrochoanal polyp tissues and control tissues. Staining intensity was graded as: 0 for no, 1 for weak, 2 for moderate, and 3 for strong. Differences in CYP and GST isoenzymes expressions between antrochoanal polyp tissues and control tissues were examined by the Mann-Whitney U test with 95% confidence level. ^a^ Mean ± standard error of the mean; ^b^ minimum and maximum staining intensity; *ratio of antrochoanal polyp tissues and control tissues; **Values of *p* < 0.05 were considered statistically significant.

**Fig. 1:**
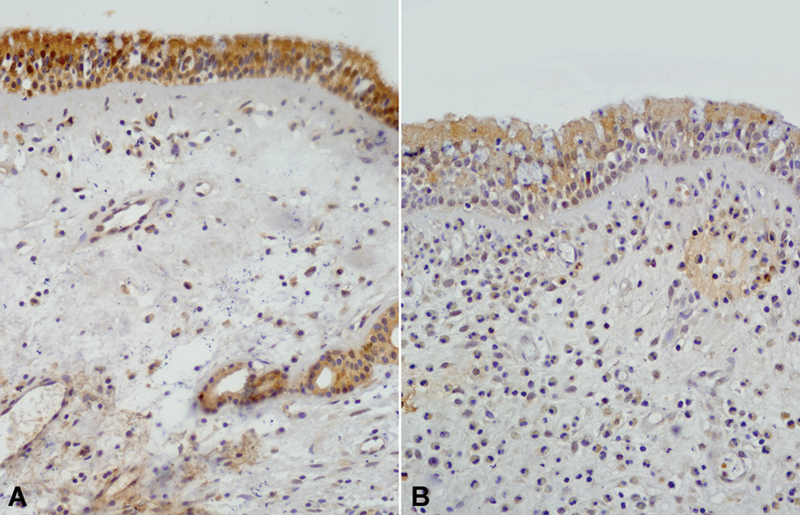
( **A** ) Control. ( **B** ) Antrochoanal polyp (ACP) patient. Patients with ACP have statistically less CYP1A1 isozyme expression than the control group (CYP1a1; original magnification: x200).

**Fig. 2:**
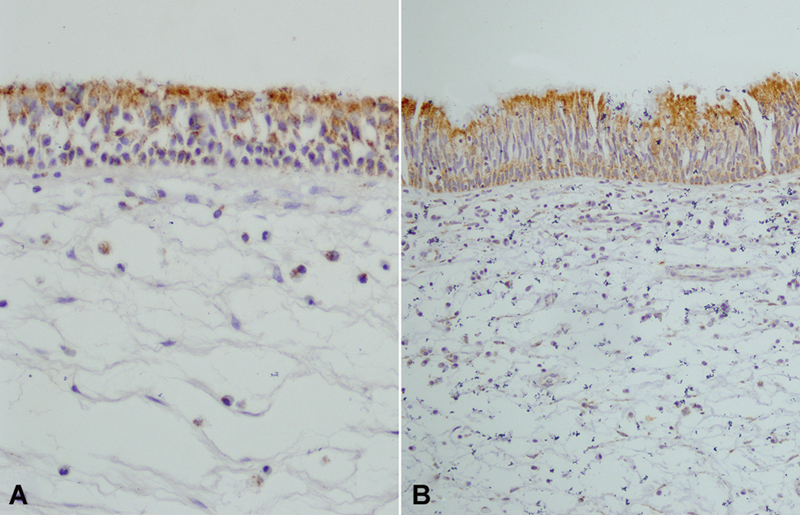
( **A** ) Control ( **B** ) Antrochoanal polyp (ACP) patient. The expression of GSTP1 in ACP patients and controls is not statistically different. (GSTP1; original magnification: x200). (Control; original magnification: x150).

Antrochoanal polyp patients had statistically less CYP1A1 isozyme expression than the control group ( *p* < 0.05) ( Fig. 1 ). Moreover GSTP1, GSTM1, and GSTA1 isozyme expressions were not different between the groups ( Figs. 2 3 4 ) ( Table 2 ). There was a positive correlation between GSTA1 and GSTM1 enzymes expressions in ACP patients (r = 0.834; *p* < 0.05).

**Fig. 3:**
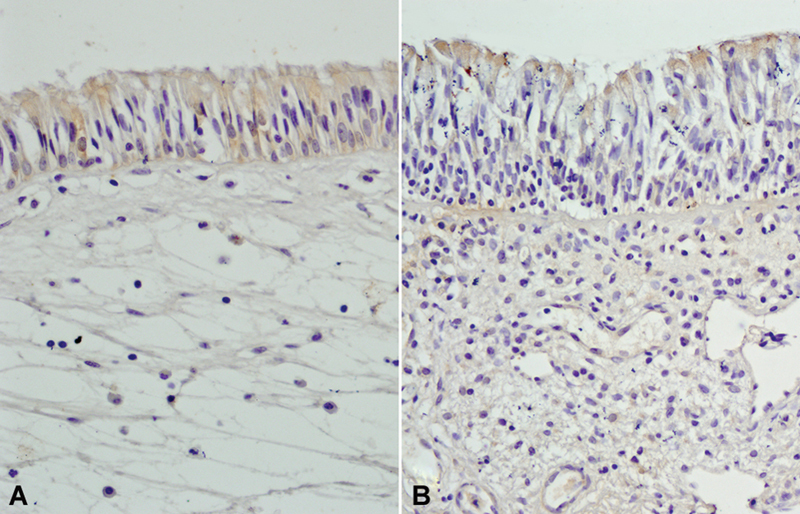
( **A** ) Control ( **B** ) Antrochoanal polyp (ACP) patient. The expression of GSTM1 in ACP patients and controls is not statistically different. (GSTM1; original magnification: x200).

**Fig. 4:**
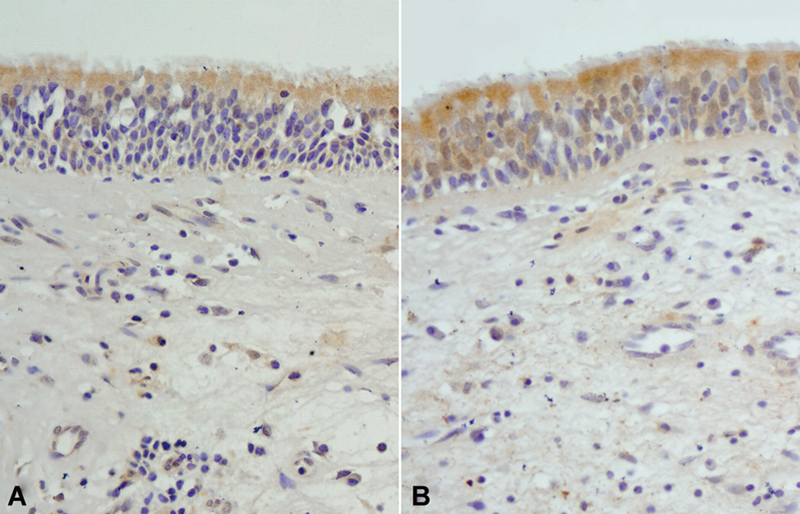
( **A** ) Control ( **B** ) Antrochoanal polyp (ACP) patient. Expression of GSTA1 in ACP patients and controls is not statistically different. (GSTA1; original magnification: x200).

## Discussion

The pathophysiology of ACP generation is still an ongoing debate, but several associated factors, such as chronic inflammation, allergy, and dental trauma, are supposed to be causes of the pathology. Overall, the most investigated pathologies are allergies and chronic inflammatory processes. Daly 15 and Ruffoli et al. 16 showed the allergic background of ACPs. Contrarily, there are also reports failing to show the role of allergy on the pathophysiology of the polyp formation. 17

Investigation of the polyp tissue revealed that the stromal layer of the polyp is highly vascular and edematous and infiltrated with plasma cells and eosinophils. 18 This finding supports the allergic status of the polyp generation. Besides, allergic nasal polyps differ from ACPs by some features. The main difference is the macroscopic characteristic feature, low eosinophil count at the tissue level, and possession of a very small amount of mucous gland within the polyp in ACPs ^19^ . Especially, these inflammatory cells (plasma cells and eosinophils) support the allergic background of ACPs formation. For this reason, the tissue destruction mechanism should be investigated.

Free radical damage, which mediated tissue injury, has been suggested as a significant reason for asthma and nasal polyp in previous reports. 6 7 8 10 19 20 Both free radical increase and decrease in antioxidant capacity at the the tissue level is related with tissue damage. Normally, this oxidant/antioxidant system balances the free radicals at the tissue level and protects them from further damage. 6 8 Free radicals are thought to destroy the ion pumps of the cell membrane and result in the impairment of the cell energy system. 7 This process results in mucosal edema and epithelial injury.

Detoxification or biotransformation is known as the system utilizing the toxic metabolites, such as free radicals, in the human body. 21 This mechanism is supplied by two integrated—phase 1 and phase 2—reactions. Phase 1 reaction refers to oxidation, reduction, and hydrolysis pathways. In this phase, the CYP enzyme system has a key role in both oxidation and reduction reactions. Polyaromatic hydrocarbons (PAHs), such as smoking, lead to increased expression of the CYP gene; therefore, the toxic metabolites generated cause cell damage. 22 Phase 2 reactions are complex conjugation and synthesis reactions. The polar products of the phase 1 reactions are combined with endogenous materials that are related to phase-2 reactions. Phase-2 reactions are achieved by conjugation (glucuronic acid, sulfate, and glutathione (GSH), methylation, and acetylation. 21 Glutathione S-transferase enzymes are very important antioxidant key products regulated by GST genes. Their role in ROS product degradation has been defined previously. The mechanism is supplied with both a direct and an indirect way of the antioxidant system.AU

Both structural, infectious diseases, allergic conditions, as well as benign and malign tumoral pathologies have been investigated previously with the GST gene family. A previous report, which was conducted with pterygium tissue, found higher expression of GSTP1 and GSTT1 than in control healthy patients. 23 Similarly, GSTA, GSTT1, and GSTM4 expressions were stronger in non-small cell lung cancer cells than normal lung epithelium. 24 Also, larynx cancer tissue had increased expression of GSTP than that of normal controls. 25 In another study, the expression of CYP1A1 and GSTP1 isozymes were found statistically significantly higher in patients who had an aortic aneurysm than in control groups. 14

The role of the GST genes in allergic diseases such as asthma and nasal polyps has been investigated before. 10 26 Glutathione S-transferase polymorphism in nasal polyps has been investigated in previous studies, and only GSTT1 levels have been found to be associated with polyp pathogenesis. 26 Both studies were about genetic analysis and tissue investigation, but the role of GST at the tissue level was not investigated. 11 26 In our study, we investigated the level of GST and CYP expressions in ACP tissue. In our findings, we have observed decreased levels of CYP1A1 isozyme in ACP. Other GST isozymes are not statistically different within the study group. The levels of GSTA1 and GSTM1 isozymes that are correlated positively in ACP patients emphasized the unchanged response in ACP pathogenesis. Our findings also are consistent with the previous reports related with gene analysis of nasal polyps. 11

Smoking habits, which can alter the inflammatory response in nasal epithelium, did not change the GST and CYP expression levels in our ACP patients. Our results agreed with those of previous reports. 27 Additionally, another study, which was conducted with structural pathologies such as aortic aneurysm, did not find any relation between ACPs and smoking habits. Recent studies have shown the relation between smoking habits and DNA adduct formation in nasal mucosal cells. 28 Smoking is related with up regulation of several subtypes of CYP genes (CYP1A1) in nasal mucosa. It also affects the genes supporting adhesion and cell cycle reactions. We have detected a decreased level of CYP1A1 levels in the ACP group. Previous reports have shown mutations in CYP1A1 in nasal mucosal cells. It is well known that CYP genes are very important in detoxification reactions in xenobiotic metabolism. Cell cycle progression increased in smokers due to toxins. This mechanism can be achieved via an increase in gene expression. 29 Epithelial cells that were exposed to smoke up regulated the oxidoreductase genes. Additionally, both aldehyde dehydrogenase and aldo-keto reductases are activated for detoxification reactions. Sridhar et al. 30 stated that smoking caused high expression of glutathione, such as GSTM1, in nasal epithelium. We have also observed high expression of CYP in nasal cells.

## Conclusion

Antrochoanal polyp is one of the most common reasons for nasal polyps, but the mechanism of pathology is still not clear. We could not find any relation with smoking habits, but we have detected a decreased CYP1A expression in ACP at the tissue level. We were not able to determine the effect of chronic inflammation on ACP formation in this study. Some consider that allergies and chronic infection are the main causes for the inflammatory process.
